# Diffusion Behavior and Fracture Mechanism at Solid–Liquid Interface of Polycrystalline Al/Mg Bimetallic System: A Molecular Dynamics Simulation

**DOI:** 10.3390/ma19050836

**Published:** 2026-02-24

**Authors:** Xiaoqiong Wang, Jingfan Cheng, Guangyu Li, Wenming Jiang, Youpeng Song, Haonan Huang, Xinyi Huang, Teng Meng, Xing Kang, Qiantong Zeng, Shan Yao, Pingkun Yao, Haytham Elgazzar

**Affiliations:** 1 School of Materials Science and Engineering, Dalian University of Technology, Dalian 116024, China; xqw@mail.dlut.edu.cn (X.W.); hhn18604082167@163.com (H.H.); cyxinyi@mail.dlut.edu.cn (X.H.); 15662792619@163.com (T.M.); xingkangx@163.com (X.K.); zqt_185@163.com (Q.Z.); yaoshan@dlut.edu.cn (S.Y.); ypingkun@dlut.edu.cn (P.Y.); 2 Hubei Engineering Research Center for Intelligent Detection and Identification of Complex Parts, Wuhan Vocational College of Software and Engineering (Wuhan Open University), Wuhan 430205, China; 2023501072@hust.edu.cn; 3 State Key Laboratory of Materials Processing and Die & Mould Technology, School of Materials Science and Engineering, Huazhong University of Science and Technology, Wuhan 430074, China; 4 China Iron and Steel Research Institute Group, Beijing 100081, China; songyoupeng@163.com; 5 Research Institute of Advanced Materials (Shenzhen) Co., Ltd., China Iron & Steel Research Institute Group, Shenzhen 518045, China; 6 The Advanced Digital Manufacturing Department, Central Metallurgical Research and Development Institute (CMRDI), Cairo 11421, Egypt; elgazzar.ha@gmail.com

**Keywords:** molecular dynamics, Al/Mg bimetal, diffusion behavior, fracture mechanism, solid–liquid interface

## Abstract

Al/Mg bimetallic composites have drawn considerable attention for their promising lightweight applications in sectors such as the aerospace and automotive industries. In these systems, the interfacial behavior critically governs the overall performance and reliability. In this research, the molecular dynamics (MD) simulation was employed to systematically study the effects of pouring temperatures (923 K, 973 K, and 1023 K) and preheating temperatures (373 K, 473 K, and 573 K) on the interfacial diffusion behavior and fracture mechanism of the polycrystalline Al/Mg bimetallic system. The results indicate that the influencing rule of pouring temperatures and preheating temperatures on the interfacial diffusion behavior is consistent. Specifically, the diffusion coefficient of Mg atoms is higher than that of Al atoms, while the diffusion distance of Al atoms is significantly greater than that of Mg atoms. As the temperature increases, the thickness of the interfacial transition layer correspondingly rises. However, the effects of these two parameters on tensile fracture behavior demonstrate notable discrepancies. Specifically, the fracture mode evolves with pouring temperature, transitioning from being mediated solely by dislocations to being co-mediated by twins and dislocations. In contrast, the fracture mechanism remains solely dislocation-controlled, regardless of the preheating temperature. In addition, all the models fractured at the interface between the diffusion layer and the Mg matrix. The optimal tensile strength of 1.850 GPa was achieved at a pouring temperature of 923 K and a preheating temperature of 473 K, representing an improvement of approximately 52% compared to the lowest value recorded in the study. This research offers significant theoretical insights for the rational optimization of preparation parameters and an in-depth understanding of fracture mechanisms in Al/Mg bimetallic systems.

## 1. Introduction

The imperative for lightweight structural materials in aerospace, automotive, and emerging sectors drives the development of advanced composites [1,2,3,4]. Achieving an optimal balance of low density, high strength, and good corrosion resistance often necessitates combining dissimilar metals. Aluminum/Magnesium (Al/Mg) bimetallic systems are a prime candidate for such applications, as they strategically integrate the complementary properties of both constituents [5,6,7]. Mg provides the lowest density among structural metals [8,9,10,11,12], while Al offers superior strength, ductility, and environmental durability [13,14,15]. This synergy makes Al/Mg bimaterials critical for components where weight saving directly translates to enhanced energy efficiency and performance.

Currently, the fabrication methods for Al/Mg bimetallic materials are primarily categorized into solid–solid and solid–liquid composite techniques [16,17]. Solid–solid compounding methods, such as roll bonding [18], explosive welding [19], extrusion bonding [20,21], and diffusion bonding [22,23], are commonly utilized. Despite their frequent use, these methods are often hindered by complex surface treatment requirements, prolonged processing times, and limitations concerning the shape and size of the materials. In contrast, the solid–liquid composite technique presents notable advantages, including freedom from shape constraints, increased efficiency in composite formation, and cost-effectiveness [24]. Among these techniques, composite casting is a particularly common and practical method. However, the interface evolution behavior during the solid–liquid composite process of Al/Mg bimetals is challenging to observe and control owing to the influence of various factors, such as the temperature and flow fields, and the complex interaction between the solid and liquid phases.

Molecular Dynamics (MD) simulations, based on classical Newtonian mechanics, can model and simulate systems ranging from a few particles to millions or even billions of particles and are capable of capturing the dynamic behavior of atoms [25,26,27]. Beyond this, MD simulations also effectively model broader materials evolution processes, such as the prediction of polycrystalline phase transformations [28,29,30]. Therefore, they hold significant advantages in studying bimetallic interfacial behavior and have become the primary means for simulating such behavior. For instance, in the studies of bimetallic systems such as Al/Cu [31,32,33,34], Al/Ti [35,36], Al/Fe [37,38,39], Al/Ni [40,41], Cu/Ni [42,43] and Cu/Fe [44,45], among others, the effects of various internal or external factors on interfacial diffusion behavior and mechanical properties have been extensively investigated. However, research on Al/Mg bimetallic systems remains relatively limited, with a lack of systematic work. In a recent study, Zhang TT et al. [46] employed the MD simulations to study atomic diffusion behavior at the bonding interface of solid–solid Al/Mg bimetal under explosive welding conditions. The results demonstrated that the diffusion coefficient of Mg atoms was higher than that of Al atoms. Li Y et al. [47] compared the mechanical properties of Al/Mg composite structures interfaced to amorphous Mg_2_Al_3_ or Mg_17_Al_12_. The results indicated that their yield strengths were lower than that of the Mg; however, the two distinct yielding mechanisms led to fractures within the Mg matrix and at the interface. Li Z et al. [48] conducted an investigation into the effects of grain size, strain rate, and the intermetallic compound layer thickness on the plastic fracture behavior of solid–solid nanocrystalline Al/Mg bimetals. Specifically, at low strain rates, the strength conforms to the Hall–Petch relationship, while at high strain rates, the elastic modulus increases with grain growth. Additionally, an optimal value for the thickness of the interlayer was identified.

It can be found that previous studies on Al/Mg bimetals have mostly focused on the solid–solid system, and treated diffusion and mechanical behaviors in isolation without combining tensile tests after diffusion. This approach not only neglects the investigation of solid–liquid systems but also fails to elucidate the underlying coupling mechanisms between diffusion and mechanical properties. To address these gaps, molecular dynamics simulations were employed in this study to systematically investigate the interfacial behavior of Al/Mg systems fabricated via the solid–liquid composite casting method. Furthermore, the influence of two key parameters, pouring temperature and preheating temperature, was systematically examined to elucidate their role in governing the interfacial fracture mechanisms. Overall, this study aimed to provide more in-depth theoretical guidance for the practical application of Al/Mg bimetals produced through composite casting.

## 2. Experimental Details and Molecular Dynamics Modeling

### 2.1. Experimental Details

In the experiments, pure Al and pure Mg were used to manufacture Al/Mg bimetals, and their compositions are listed in Table 1. In the solid–liquid composite model, pure Al rods of a diameter of 15 mm and a height of 110 mm were used as solid inserts. The inserts were sequentially polished with 220, 400, 1000, and 2000 grit silicon carbide sandpaper to remove the oxide layer, followed by ultrasonic cleaning in anhydrous ethanol for 10 min. The cleaned Al inserts were then preheated to 100, 200, and 300 °C in a drying oven. Pure Mg was melted and held at 650, 700, and 750 °C in a resistance furnace. Five sets of solid–liquid composite samples were prepared: samples with Al inserts preheated to 200 °C and Mg poured at 650, 700, and 750 °C and samples with Mg poured at 650 °C and Al inserts preheated to 100 and 300 °C.

For the solid–solid composite model, pure Al and Mg plates with dimensions of 50 mm × 50 mm × 5 mm were used. The plates were preheated to 200 and 500 °C and held for 6 h. Subsequently, the preheated plates were clamped together and held at 400 °C for 24 h to facilitate bonding.

After the casting, the samples were sectioned by wire cutting and ground sequentially with 80, 200, 400, 1500, and 2000 grit sandpapers. All prepared samples were subsequently polished with 1.5 μm diamond paste and then etched with a 4% nitric acid alcohol solution for 5 s to remove surface machining marks. The microstructure and chemical composition of the Al/Mg bimetallic composites were analyzed using a JSM IT800 scanning electron microscope (SEM) equipped with energy-dispersive X-ray spectroscopy (EDS).

### 2.2. Molecular Dynamics Modeling

**Figure 1:**
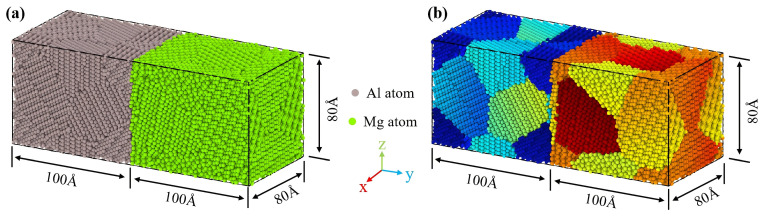
Al/Mg polycrystal diffusion model: (**a**) Atomic configuration, with Al atoms in gray and Mg atoms in green, (**b**) different grains are distinguished by different colors.

The initial atomic structure model was constructed using the open-source Atomsk software (version b0.13.1) [49,50]. A polycrystalline Al/Mg bimetallic model, composed of pure Al and pure Mg, with the size of 80 Å (X) × 200 Å (Y) × 80 Å (Z), was constructed, in which the numbers of Mg atoms and Al atoms were 38,518 and 27,537, as presented in Figure 1. Periodic boundary conditions were applied on the two pairs of opposite surfaces perpendicular to the X and Z axes, respectively, while the surfaces perpendicular to the Y axis were set as free boundaries. The initial atomic velocities obey the Maxwell speed distribution. Newton’s equations of motion for atoms were numerically integrated using the Velocity Verlet algorithm with a timestep of 1 fs.

The Finnis–Sinclair interatomic potential proposed by Mendelev et al. [51] was employed in this work. The Finnis–Sinclair potential is established on the foundation of the second-moment approximation within tight-binding theory. Moreover, valuable research on the mechanical properties of Al/Mg materials simulated using MD with the Finnis–Sinclair potential has been reported [52,53,54]. Further extending its applicability, the same potential has also been utilized to investigate the thermal behavior of Mg-Al systems under extreme conditions, such as in the simulation of the melting process of Mg-Al nanolayers at a heating rate of 1000 K/ps, including ultra-high-temperature states up to 1832 K [55], and in studies of the structural and dynamic properties of Mg-Al alloys in the liquid state at 1500 K [56]. Therefore, employing this interatomic potential to model the mechanical behavior of interfacial diffusion in Al/Mg bimetal systems is well-founded.

In this research, the Large-scale Atomic/Molecular Massively Parallel Simulator (LAMMPS, version 3Nov2022-MPI) MD software was used to simulate the interfacial diffusion behavior and uniaxial tensile behavior of the Al/Mg bimetals [57,58,59]. Following an initial equilibration at 1 K for 40 ps to achieve atomic-scale relaxation, the Al substrate was heated to predetermined preheating temperatures (373 K, 473 K, and 573 K) using an isothermal–isobaric (NPT) ensemble, followed by isothermal holding within a canonical (NVT) ensemble to investigate the temperature-dependent interfacial diffusion kinetics. Subsequently, the Mg substrate was thermally elevated to pouring temperatures (923 K, 973 K, and 1023 K) using an NPT ensemble until complete melting, followed by holding in the NVT ensemble, whereupon the molten Mg was brought into contact with solid Al to simulate the pouring process, thereby probing the influence of pouring temperature on interfacial interdiffusion. The complete melting of Mg atoms at different pouring temperatures was identified by common neighbor analysis (CNA), characterized by the disappearance of the characteristic HCP structure and its transition to another state. The Al/Mg bimetal system was then subjected to a diffusion simulation for 2 ns under a microcanonical (NVE) ensemble to investigate the atomic interdiffusion behavior. The resultant Al/Mg diffusion model was quenched to 300 K and thermally equilibrated using a Nose–Hoover thermostat within an NPT ensemble. Uniaxial tensile deformation was then imposed along the Y-axis at a strain rate of 10^10^ s^−1^ on Al/Mg bimetals formed at various pouring temperatures or preheating temperatures. Atomic trajectory and structural analysis, including CNA and other post-processing procedures, were performed using the Open Visualization Tool (OVITO) [60,61,62].

It should be noted that while the strain rates accessible to MD simulations are higher than those in real experiments, the primary objective of this study was not to predict quantitative mechanical properties for direct experimental comparison. Instead, it was focused on extracting fundamental, atomic-scale trends in diffusion behavior and identifying the mechanistic transitions in deformation (i.e., from dislocation slip to twinning) that are governed by temperature parameters.

## 3. Results

### 3.1. Experimental Results

**Figure 2:**
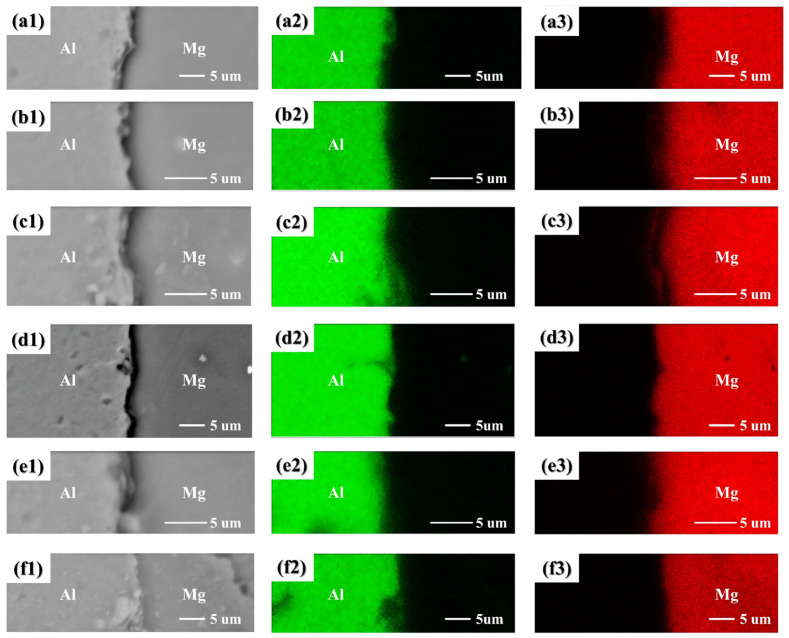
Typical morphologies and EDS maps of the Al/Mg interfaces: (**a1**–**a3**) 473 K(Al)−773 K(Mg); (**b1**–**b3**) 473 K(Al)–923 K(Mg); (**c1**–**c3**) 473 K(Al)–973 K(Mg); (**d1–d3**) 473 K(Al)–1023 K(Mg); (**e1**–**e3**) 373 K(Al)–923 K(Mg); (**f1**–**f3**) 573 K(Al)–923 K(Mg).

Figure 2 presents the microstructure of the bimetallic interface under different conditions. As observed in Figure 2(a1–f1), an interface was formed between the Al matrix and Mg matrix, which remained free of intermetallic compounds despite variations in pouring temperatures and preheating temperatures. The EDS mapping results (Figure 2(a2–f3)) confirmed the formation of a diffusion zone but definitively ruled out the formation of any intermetallic compounds (IMCs).

### 3.2. Diffusion Behavior

#### 3.2.1. Difference in Diffusion Behavior Between the Solid–Solid and Solid–Liquid Systems

**Figure 3:**
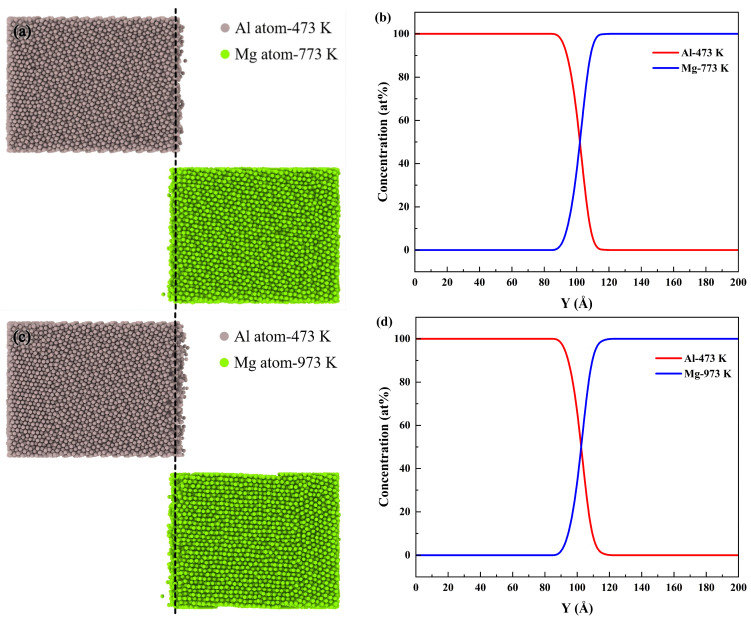
Atomic configurations and concentration profiles in different systems: (**a**) Configurations of the cross section in solid–solid system; (**b**) Y direction concentration of Al and Mg atoms in solid–solid system; (**c**) configurations of the cross section in solid–liquid system; (**d**) Y direction concentration of Al and Mg atoms in solid–liquid system.

The diffusion behaviors at different interfaces were compared by establishing two distinct Al/Mg systems. In the solid–solid model, Al and Mg were maintained in solid states at 473 K and 773 K, respectively, while the solid–liquid model consisted of solid Al at 473 K and liquid Mg at 973 K. A comparative analysis of the diffusion behavior is presented in Figure 3. Figure 3a,c revealed that the number of diffusing Mg atoms and the distance they crossed the initial interface showed no significant change between the two systems. In contrast, Al atoms exhibited a markedly greater diffusion distance in the latter than in the former. This enhancement is attributed to the increased temperature, which intensifies atomic mobility, resulting in both longer diffusion distances and a higher number of mobile atoms in the solid–liquid system.

In MD simulations, a region with a diffusing atom concentration typically higher than 5% (by atomic fraction) is considered a transition layer [63,64]. To further quantify the interdiffusion, the diffusion layer thickness was determined from the atomic concentration profiles along the Y−axis, as shown in Figure 3b,d. The thicknesses of the interface transition layers in both solid–solid and solid–liquid systems were determined to be approximately 18.53 Å and 19.13 Å, respectively. This indicated that the transition layer thickness increased with increasing temperature. Higher temperatures reduce the diffusion activation barriers for Al/Mg bimetals, leading to an increase in the diffusion coefficient and consequent growth of the transition layer.

In general, the diffusion coefficient is commonly used to express the diffusion capacity. The positions of atoms are constantly changing at different moments, so the diffusion coefficient is often predicted by calculating Mean Square Displacement (MSD) in MD. The calculation formula of MSD can be expressed as [65,66,67]:

(1) $$MSD=\frac{1}{N}\overset{N}{\underset{i=1}{\sum }}<{\left|r_{i}\left(t\right)-r_{i}\left(0\right)\right|}^{2}>$$

where *r_i_*(*t*) is the position vector of the atom at t time. According to the diffusion law, the diffusion coefficient *D* can be obtained from [68,69]:

(2) $$D=\underset{t\to \infty }{lim}\frac{1}{2Nt}<{\left|r\left(t\right)-r\left(0\right)\right|}^{2}>$$

where *N* is the dimension of the simulation system, and *N* is 3 in this system.

**Figure 4:**
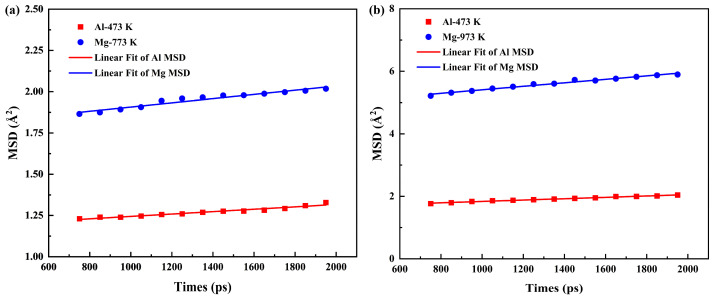
MSD in Al/Mg bimetallic systems: (**a**) Solid–solid system; (**b**) solid–liquid system.

Figure 4 presents the diffusion coefficients along the Y direction for Al atoms and Mg atoms in the two systems. It can be seen that the diffusion coefficients of the solid–liquid diffusion system were much higher than those of the solid–solid diffusion system. Moreover, Mg atoms exhibited higher diffusion coefficients than Al atoms. However, the simulations revealed that Al atoms achieved longer migration distances. This indicates that the diffusion distance is influenced by the nature of the matrix structure, not just the diffusion coefficient, which can be attributed to the following factors.

**Figure 5:**
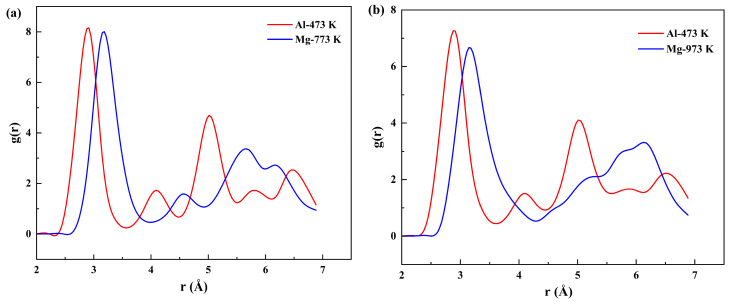
RDF of Al and Mg atoms: (**a**) Solid–solid diffusion; (**b**) solid–liquid diffusion.

Firstly, structural ordering within atomic systems is conventionally characterized using the radial distribution function (RDF) in MD simulations [70,71,72,73]. The RDF profiles in Figure 5 present the results for the Al and Mg atoms in different systems. The peak positions in the RDF correspond to the specific coordination shells. The first major peak represents the first coordination shell, and its sharpness and high intensity indicate a high degree of short−range order in the atomic arrangement of the material.

An increase in temperature resulted in a marked broadening and a decrease in the intensity of the RDF peaks for both Al and Mg atoms. This phenomenon is attributed to enhanced atomic thermal vibrations, which cause atoms to deviate from their ideal lattice positions, thereby disrupting the long−range order. Notably, within the same diffusion system (solid–solid or solid–liquid), Mg atoms exhibited consistently lower peak intensities than Al atoms. This indicates a stronger local structural disorder around the Mg atoms.

This structural disparity has critical implications for the local structural stability and diffusion behavior. First, the lower structural stability on the Mg side promotes the formation of vacancies and defects, facilitating the diffusion of Al atoms into the Mg matrix. Second, the higher melting point of Al indicates stronger metallic bonds that are more difficult to break, thereby hindering the diffusion of Mg atoms into the Al lattice. Third, the smaller atomic radius of Al (0.143 nm) compared to that of Mg (0.160 nm) allows Al atoms to diffuse more readily into the larger Mg lattice. Consequently, the synergistic effect of these factors led to a greater diffusion distance for Al atoms than for Mg atoms, further validating the previous findings.

**Figure 6:**
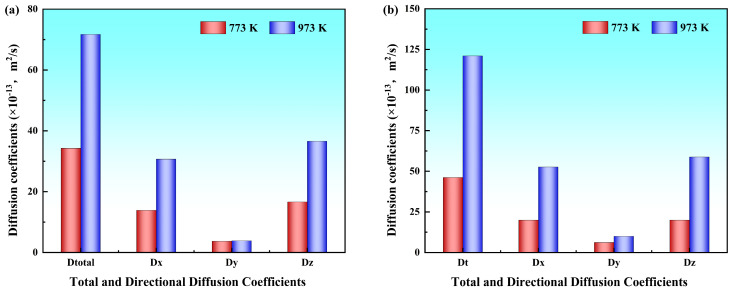
Diffusion coefficients in different Al/Mg bimetallic systems: (**a**) Al atoms; (**b**) Mg atoms.

Beyond the Y directional analysis, the diffusion coefficients along the X and Z directions and the total coefficients were quantitatively analyzed, presenting specific numerical values provided in Table 2. To more clearly illustrate the variation trends with temperature under both solid–solid and solid–liquid systems, the results are presented in Figure 6. The analysis revealed that Al and Mg atoms exhibited significant diffusion anisotropy across different directions; the diffusion coefficient in the Y direction was notably lower than that in the X and Z directions, whereas the values along the X and Z directions were comparable. This showed that atomic diffusion perpendicular to the interface (i.e., along the Y direction) encountered greater resistance, giving rise to a distinct anisotropic diffusion behavior.

This pronounced diffusion anisotropy is a key to understanding the apparent paradox of higher diffusion coefficients of Mg atoms versus greater diffusion distances of Al atoms. Although the Mg atoms exhibit higher diffusion coefficients owing to their more open lattice structure and weaker atomic bonding, they readily undergo reverse migration or random walk between vacancies owing to the lack of a well−defined directional driving force and may even become trapped by defects such as grain boundaries and dislocations at the interface, resulting in sluggish net displacement accumulation along the Y direction. In contrast, despite their lower diffusion coefficients, the smaller atomic radius of Al facilitates migration along low−energy pathways, such as grain boundaries and dislocation lines. Although these paths exhibit higher activation energies that limit the diffusion coefficient, they favor directed long−range diffusion. Consequently, the synergy between diffusion anisotropy and element−specific migration behaviors leads to the co−existence of higher diffusion coefficients in Mg and greater penetration distances in Al.

The simulated diffusion asymmetry, where Al atoms penetrate deeper into the Mg side, without forming a continuous IMC layer, was corroborated by the accompanying experimental study. Microstructural and compositional analyses (via SEM/EDS) of Al/Mg composite castings, fabricated under conditions mirroring the simulation parameters, confirmed the presence of a diffusion zone without detectable continuous IMC layers at the interface.

#### 3.2.2. Diffusion Behavior at Different Pouring Temperatures

Experimental results indicate that pouring temperature is one of the most significant factors affecting the solid–liquid diffusion behavior [74,75]. Hence, this section simulated the solid–liquid diffusion behavior of Al/Mg bimetals under varying pouring temperatures.

**Figure 7:**
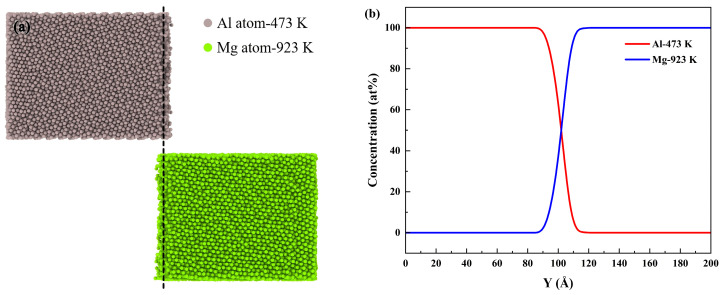

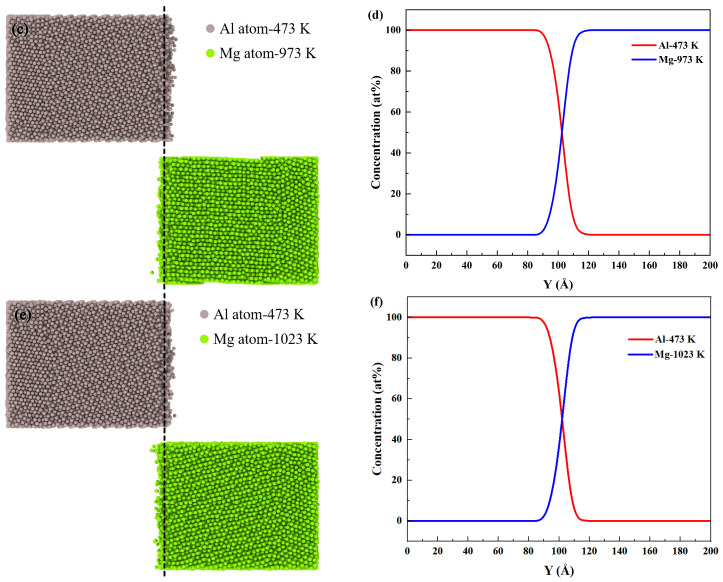
Atomic diffusion and concentration profile of Al/Mg bimetal systems at different pouring temperatures: (**a**,**c**,**e**) Configurations of the cross section at 923 K, 973 K and 1023 K, respectively; (**b**,**d**,**f**) concentration profile of Al and Mg atoms along the Y direction at 923 K, 973 K and 1023 K, respectively.

Figure 7a,c,e illustrate the atomic diffusion behavior observed at a preheating temperature of 473 K under varying Mg pouring temperatures (923 K, 973 K and 1023 K). As depicted in Figure 7, the atomic diffusion at the interface increased with the rise in pouring temperature, leading to enhanced mutual diffusion of Al and Mg atoms. Furthermore, asymmetric diffusion between Al and Mg atoms was observed, as the interfacial diffusion process was primarily driven by the diffusion of Al atoms into the Mg side. The underlying reasons for this phenomenon were comprehensively explained in the preceding section. Figure 7b,d,f illustrate the atomic concentration profiles along the Y direction for the Al/Mg bimetal at different pouring temperatures. Across all pouring temperatures, the pattern of atomic concentration change at the interface remained consistent.

**Figure 8:**
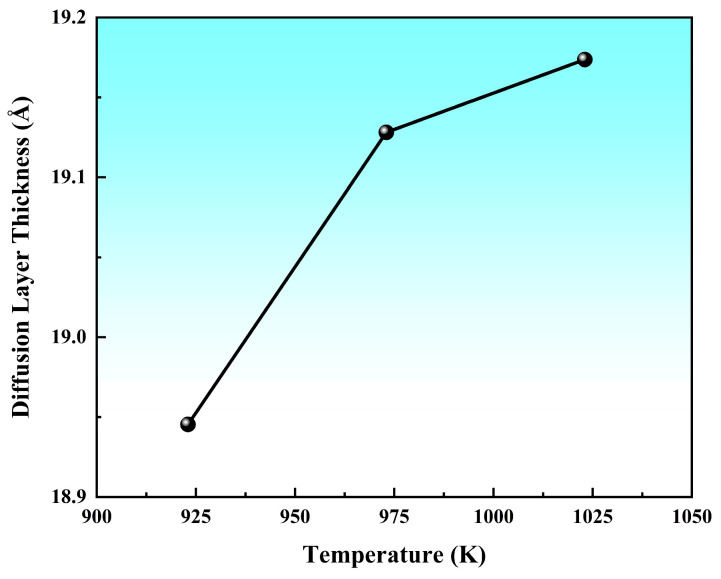
Diffusion layer thickness as a function of pouring temperature.

Figure 8 depicts the variation curve of the transition layer thickness in the Al/Mg bimetal as a function of pouring temperature. The thickness of the transition layer increased with rising pouring temperatures. When the temperature was below 973 K, the thickness of the interface transition layer increased sharply with temperature. However, once the temperature surpassed 973 K, the growth rate of the thickness decelerated significantly. This deceleration is attributed to the increased diffusion distance caused by the thickening of the transition layer. Consequently, the diffusion flux diminished as the thickness increased, leading to a gradual slowdown in the growth rate of the interfacial layer thickness.

**Figure 9:**
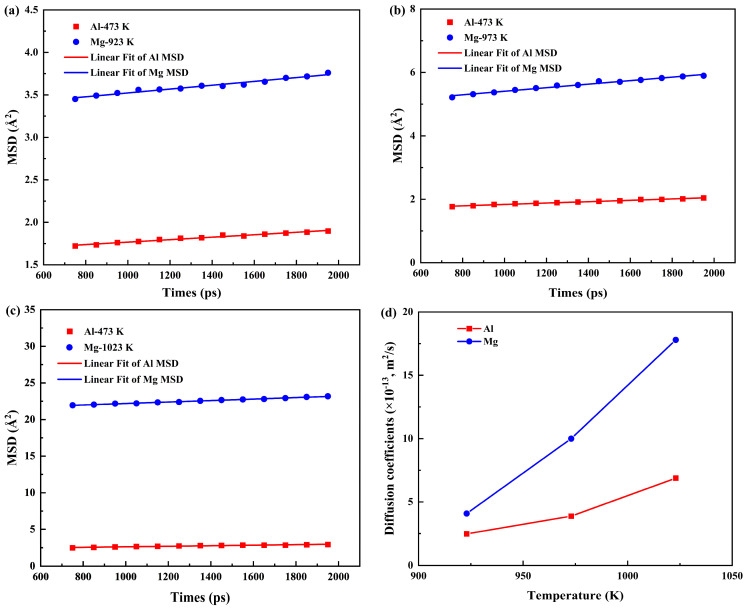
The MSD and calculated diffusion coefficients of Al/Mg bimetals: (**a**−**c**) MSD at various pouring temperatures of 923 K, 973 K, and 1023 K; (**d**) diffusion coefficients as a function of pouring temperature.

The diffusion coefficients at corresponding temperatures were obtained by linearly fitting the MSD curves of Al and Mg atoms along the Y direction in the Al/Mg bimetallic system under various pouring temperatures. Further analysis revealed a notable increase in diffusion coefficients with rising pouring temperature, as detailed in Figure 9. Specifically, the MSD values of both Al and Mg atoms showed a continuous upward trend at higher pouring temperatures, which reflected intensified atomic thermal motion. Consequently, in accordance with the Einstein diffusion relation, this enhancement resulted in strengthened diffusion capacity.

**Figure 10:**
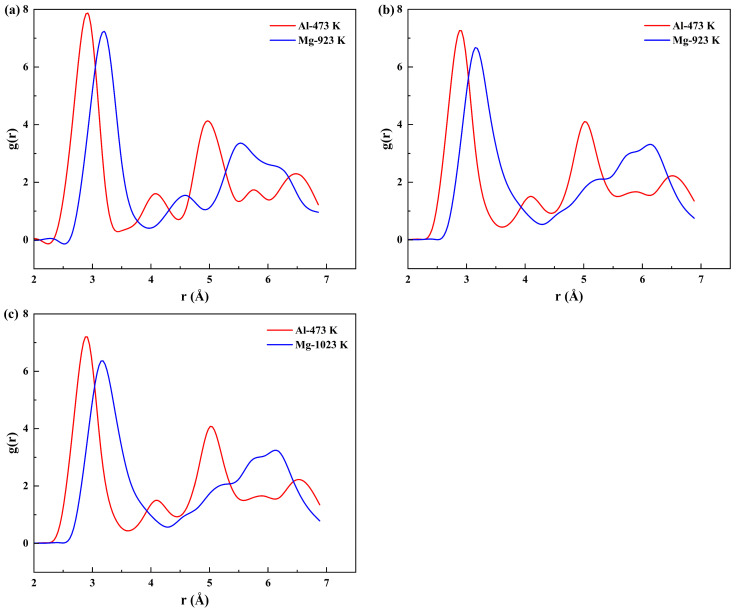
RDF of Al and Mg atoms at different pouring temperatures: (**a**) 923 K; (**b**) 973 K; (**c**) 1023 K.

The RDFs for the Al/Mg bimetallic systems at different pouring temperatures are presented in Figure 10. It was found that as the pouring temperature increased, the primary RDF peaks for both Al and Mg underwent significant broadening, accompanied by a decrease in peak intensity. The broadening of the first coordination shell peak indicates a wider distribution of distances between an atom and its nearest neighbors, providing direct evidence of increased structural disorder and reduced local structural stability. The decrease in the peak intensity reflects a lower atomic packing density within the first coordination shell.

Notably, within the same system, the peak values of the RDF on the Al side were significantly higher than those on the Mg side. This confirms that the Al lattice exhibits superior thermal stability compared to the Mg lattice at high temperatures. The stronger bonding in the Al lattice enables Al atoms to maintain a higher degree of short−range order during high−temperature diffusion, thereby hindering the diffusion of Mg atoms into it more effectively. Conversely, the rapidly deteriorating local structural stability on the Mg side with increasing temperature creates preferential pathways for Al atom diffusion, which explains the observed asymmetric diffusion behavior from an atomic structural perspective.

**Figure 11:**
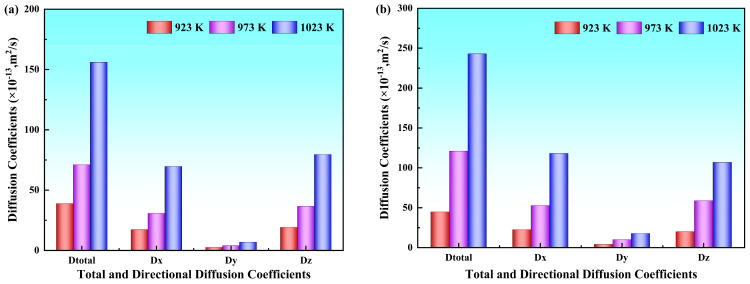
Diffusion coefficients at different pouring temperatures: (**a**) Diffusion coefficients of Al atoms at different pouring temperatures; (**b**) diffusion coefficients of Mg atoms at different pouring temperatures.

The total diffusion coefficients of Al and Mg atoms, along with their directional components along the X, Y and Z directions at different pouring temperatures, were calculated using the Einstein diffusion equation. The variation trends of these diffusion coefficients with the pouring temperature are illustrated in Figure 11, and the corresponding numerical values are listed in Table 3. The results demonstrate that the diffusion coefficients of Mg atoms are systematically higher than those of Al atoms in all three directions at all temperatures. The diffusion coefficients of both Al and Mg atoms along the X and Z directions were markedly higher than those along the Y direction, and all the diffusion coefficients exhibited progressive enhancement with increasing pouring temperature.

#### 3.2.3. Diffusion Behavior at Different Preheating Temperatures

Given that the preheating temperature is a critical factor influencing interfacial bonding [76], its effect on mitigating thermal stress to prevent cracking and facilitating controlled atomic diffusion for robust metallurgical bonding was systematically examined and is presented in this section.

**Figure 12:**
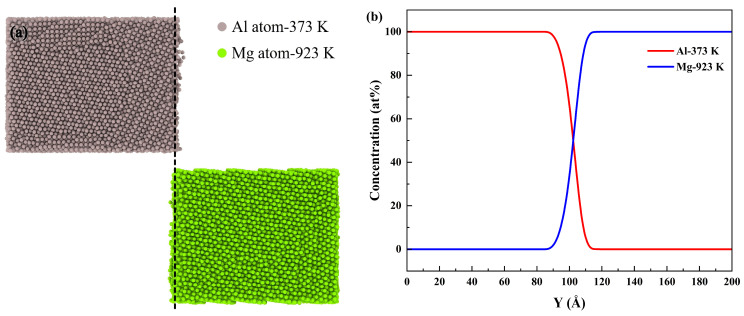

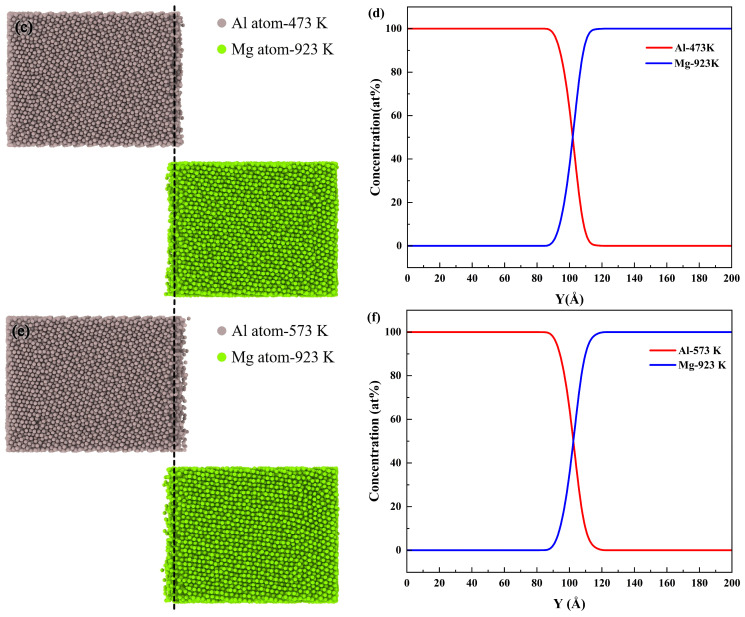
Atomic diffusion and concentration profile of Al/Mg bimetallic systems at different preheating temperatures: (**a**,**c**,**e**) Configurations of the cross section at 373 K, 473 K and 573 K, respectively; (**b**,**d**,**f**) concentration profile of Al and Mg atoms along the Y direction at 373 K, 473 K and 573 K, respectively.

Figure 12 illustrates the atomic diffusion behavior at a pouring temperature of 973 K, considering various preheating temperatures (373 K, 473 K, and 573 K) for the Al side. As the preheating temperature of Al increased from 373 K to 573 K, the atomic diffusion exhibited a pattern consistent with that depicted in Figure 12a,c,e: with the rise in preheating temperature, the diffusion distances of both Al and Mg atoms increased. This consistency is attributed to the equivalent regulatory mechanism of temperature on diffusion kinetics. Specifically, an increase in either the pouring temperature or the preheating temperature reduces the diffusion activation energy, thereby enhancing the atomic diffusion coefficients. Consistent with the trend observed under varying pouring temperatures, the atomic concentration profiles in Figure 12b,d,f demonstrated a similar interdiffusion pattern across different preheating temperatures. This consistency further confirmed that an increase in temperature, regardless of whether it was the pouring or preheating temperature, enhanced atomic interdiffusion.

**Figure 13:**
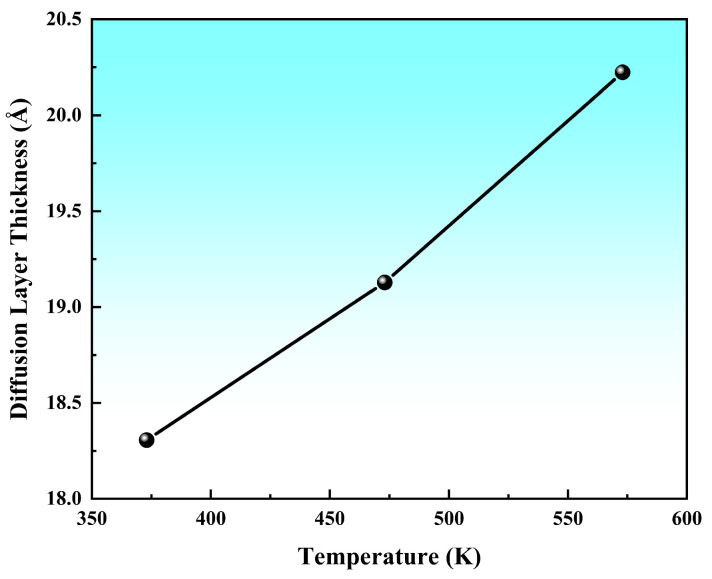
The diffusion layer thickness as a function of preheating temperature.

Figure 13 illustrates the variation curve of the interfacial transition layer thickness with the preheating temperature. The data revealed that at a fixed pouring temperature of 973 K, the diffusion layer thickness exhibited a near−linear increase as the preheating temperature rose from 373 K to 573 K. At the lowest preheating temperature (373 K), the diffusion layer remained extremely thin, even thinner than that observed in solid−state diffusion processes, with a thickness of approximately 18.31 Å. Furthermore, comparative analysis demonstrated that the preheating temperature exerted a greater influence on the diffusion layer thickness than the pouring temperature. The key result here was that the transition layer thickness, quantified from these profiles, showed a clear positive correlation with the preheating temperature.

**Figure 14:**
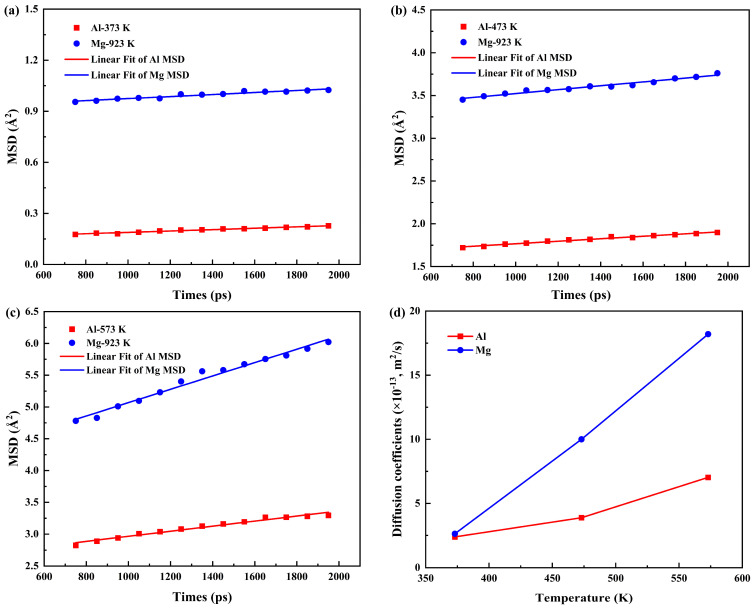
MSD and calculated diffusion coefficients of Al/Mg bimetals: (**a**–**c**) MSD at various preheating temperatures of 373 K, 473 K, and 573 K; (**d**) diffusion coefficients as a function of preheating temperature.

To further elucidate the influence of temperature, the diffusion coefficients of Al and Mg atoms were evaluated under varying preheating temperatures, as shown in Figure 14. Consistent with the earlier observations in both solid–solid and solid–liquid states, an increase in preheating temperature resulted in enhanced atomic diffusion for both species. Notably, the diffusion coefficients of Mg atoms consistently surpassed those of Al under all preheating conditions, reaffirming the trend established across different phase states and pouring temperatures.

**Figure 15:**
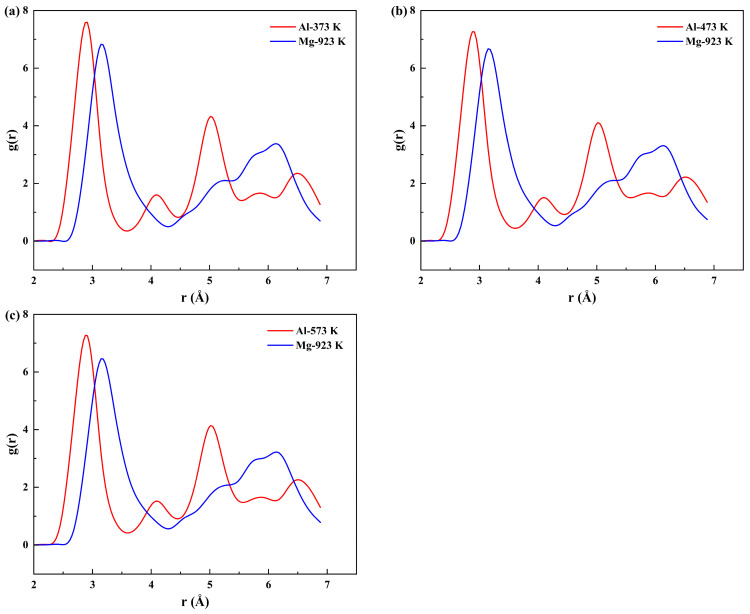
RDF of Al and Mg atoms at various preheating temperatures: (**a**) 373 K; (**b**) 473 K; (**c**) 573 K.

Figure 15 shows the RDF of Al and Mg at different preheating temperatures. The evolution of the RDF in the Al/Mg bimetal system reveals a universal pattern: the RDF trends of the Al and Mg atoms exhibit highly consistent behavior across both the preheating and pouring temperature regimes. Specifically, increasing temperature leads to continuous broadening and intensity attenuation of the RDF peaks for all coordination shells, particularly the first shell.

This systematic variation demonstrates that the fundamental mechanism of increasing the preheating temperatures and pouring temperatures is to reduce the local structural stability of the system by enhancing atomic thermal motion. The consistently lower RDF peak intensities for Mg reaffirm that its lattice is thermodynamically less stable than that of Al and more susceptible to developing structural defects under thermal agitation. This inherent local structural instability is the structural root cause for the Mg matrix being the weaker side and the primary atomic migration zone during the interfacial diffusion.

**Figure 16:**
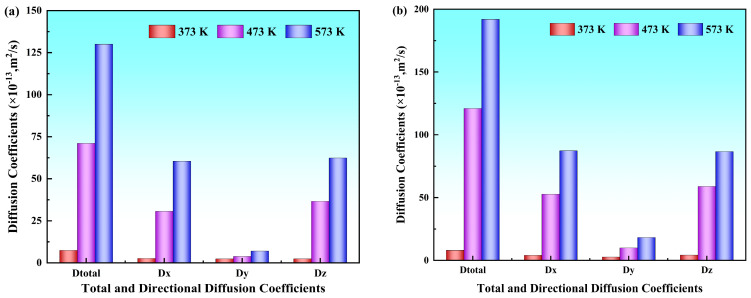
Diffusion coefficients at different preheating temperatures: (**a**) Diffusion coefficients of Al atoms at different preheating temperatures; (**b**) diffusion coefficients of Mg atoms at different preheating temperatures.

The diffusion coefficients of both Al and Mg atoms across all preheating temperatures demonstrate consistent regularity identical to that in pouring temperature systems: Mg atoms exhibit systematically higher diffusion coefficients than Al atoms along the X, Y, and Z directions, with X and Z directional components being approximately 2–3 times greater than the Y directional component. All diffusion coefficients progressively increase with rising temperature, as listed in Table 4. The variation trend is illustrated in Figure 16.

These results showed that preheating temperature provides a stronger effect on the thickness of the interfacial transition layer than the pouring temperature.

### 3.3. Tensile Fracture Behavior

#### 3.3.1. Effects of Pouring Temperatures on Tensile Fracture Behavior

To investigate the influence of pouring temperature on the tensile fracture mechanisms, the models were subjected to uniaxial tensile testing following the diffusion process.

**Figure 17:**
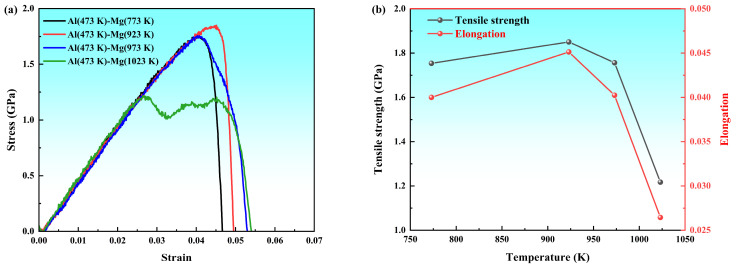
Tensile properties of Al/Mg diffusion systems at various pouring temperatures: (**a**) Stress–strain curves; (**b**) ultimate tensile strength and elongation.

Figure 17a presents the uniaxial tensile stress–strain curves of the Al/Mg bimetallic system prepared at different pouring temperatures. With increasing pouring temperature (923 K, 973 K, and 1023 K), the material exhibited a decline in both tensile strength and elongation, while the elastic modulus showed no temperature dependence. The overall trend of the curves for solid–liquid systems at 923 K and 973 K aligned with that of the 773 K solid–solid system, implying comparable diffusion−controlled interfacial bonding and fracture mechanisms across this range. The solid–solid system, however, displayed slightly lower tensile strength due to inadequate atomic diffusion and weaker metallurgical bonding from its lower processing temperature. A distinct fracture pattern emerged at 1023 K, where the curve exhibited one fluctuation prior to reaching the peak stress, which was then followed by a sharp drop to zero. This phenomenon shows staged cracking or local plastic mismatch during interfacial layer failure. It directly evidences the detrimental effect of excessive temperature on interfacial stability.

All tensile tests were performed at a constant temperature of 300 K, regardless of the pouring temperature used during the specimen preparation. Therefore, although the elastic modulus is inherently temperature−sensitive, its measured value in this study did not vary with the pouring temperature because the testing temperature was strictly controlled. This ensured that the observed mechanical behavior reflected the influence of the pouring temperature alone, without the confounding effect of the testing temperature.

Figure 17b illustrates the relationship between the mechanical properties and pouring temperature to clarify trends in tensile strength and elongation. It can be observed that both properties followed a similar trajectory, initially rising and then declining with increasing pouring temperature. The bimetal poured at 923 K exhibited optimal performance, achieving a peak tensile strength (1.850 GPa) and an elongation (4.51%). In contrast, the sample poured at 1023 K showed the poorest properties, with the optimal specimen displaying a 12.65% higher tensile strength and 52% greater elongation than the lowest−performing case.

**Figure 18:**
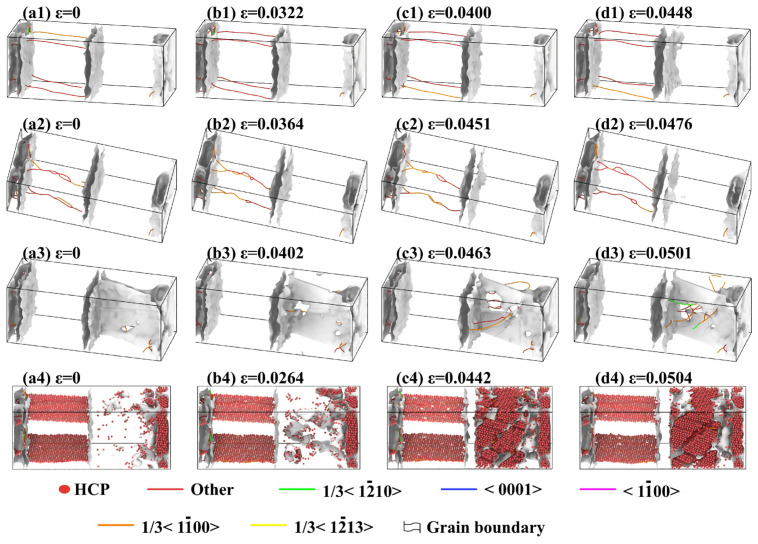
Dislocation analysis of Al/Mg bimetals at various pouring temperatures: (**a1**–**d1**) 773 K; (**a2**–**d2**) 923 K; (**a3**–**d3**) 973 K; (**a4**–**d4**) 1023 K.

Dislocation analysis (DXA) was performed to investigate the fracture mechanism of Al/Mg systems, as shown in Figure 18. Critically, both the solid–solid diffusion systems and solid–liquid diffusion systems (poured at 923 K) exhibited nearly identical DXA results. The analysis revealed that a certain dislocation density already existed in the Al matrix from initial straining. During the subsequent tensile process, no significant multiplication of dislocations was observed. This was attributed to residual stresses from thermal expansion mismatch during diffusion and cooling. Those stresses caused dislocations to be pinned at interfacial obstacles like grain and phase boundaries upon formation. The pinned dislocations remained inactive as dislocation sources under room−temperature tension and could not participate in slip, leading to macroscopic brittle fracture.

Figure 18(a3,b3) show that the model underwent purely elastic deformation without dislocation activity at strains below 4.02%. As revealed in Figure 18(c3), dislocation nucleation and multiplication commenced in the Mg matrix once the localized interfacial stress exceeded the fracture limit, initiating microcracks. Their rapid propagation along the weak interface triggered a major stress drop. Subsequent stress release enabled the crack tip to penetrate the Mg matrix, creating a plastic dissipation zone through dislocation slip that was accompanied by a gradual stress decline. Although the crack partially advanced into the matrix, the interface persisted as the preferred low−energy path, resulting in final interfacial fracture.

Analysis of the initial crystal structure at a pouring temperature of 1023 K revealed purely elastic deformation without intragranular dislocation formation at strains below 2.64%, as shown in Figure 18(a4,b4). Once the strain exceeded this threshold, deformation twins initiated and propagated in Figure 18(c4). Further strain increase was observed to restrict twin growth, as shown in Figure 18(d4); this process was accompanied by the generation of numerous dislocations, which subsequently moved and aggregated at the twinning plane. The primary reason for the lowest tensile strength in the sample poured at 1023 K was attributed to the difference in deformation mechanisms. Microstructural analysis indicated that deformation was dominated by the deformation twins. As the critical stress for twin nucleation is inherently lower than for dislocation slip, this led to the observed reductions in both yield and tensile strength. It is worth mentioning that the current simulation models represent an idealized, contamination−free interface between the pure Al and pure Mg. In practical experiments, factors such as surface oxides, impurity elements, and the inevitable formation of intermetallic compounds (IMCs) like Mg_17_Al_12_ will significantly influence interfacial strength and fracture behavior. The value of the present study lies in isolating the intrinsic effects of core thermal parameters, melting and preheating temperatures on atomic diffusion and deformation mechanisms. The determined mechanisms, such as the detrimental shift towards twin−dominated fracture at excessive melting temperatures, provide a fundamental guideline. In practice, the optimal temperature window suggested here (923 K melt, 473 K preheat) may require adjustment to balance beneficial diffusion against the kinetics of brittle IMC formation, which is an essential consideration for subsequent experimental work.

**Figure 19:**
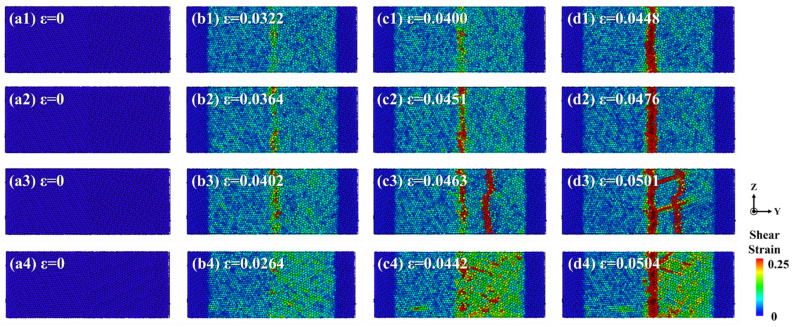
The strain distributions of Al/Mg bimetals at various pouring temperatures: (**a1**–**d1**) 773 K; (**a2**–**d2**) 923 K; (**a3**–**d3**) 973 K; (**a4**–**d4**) 1023 K.

In this study, the atomic strain analysis method was employed to visualize and track the shear strain distribution and evolution in solid–solid diffusion systems and solid–liquid diffusion systems during the tensile testing. Figure 19 illustrates the strain distribution characteristics within the Al/Mg diffusion systems. As the strain increased, the heterogeneous interface between the diffusion layer and the Mg matrix intensified stress concentration at the interface, leading to the initiation and propagation of micro−cracks at this location, ultimately resulting in fracture.

#### 3.3.2. Effects of Preheating Temperatures on Tensile Fracture Behavior

**Figure 20:**
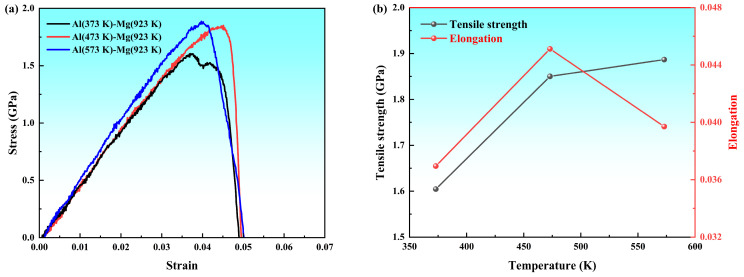
Tensile properties of Al/Mg diffusion systems at various preheating temperatures: (**a**) Stress–strain curves; (**b**) ultimate tensile strength and elongation.

The uniaxial tensile stress–strain curves of the Al/Mg bimetallic diffusion system at different preheating temperatures are shown in Figure 20a. The stress–strain curve at a preheating temperature of 473 K exhibited a trend similar to that at 573 K, with comparable tensile strength observed in both structures. However, the model at 573 K demonstrated a higher elastic modulus.

The tensile strength and elongation at different preheating temperatures can be obtained, as shown in Figure 20b. With increasing preheating temperature, the tensile strength gradually increased. Obviously, the elongation of the Al/Mg bimetal initially increases and then decreases. Considering both tensile strength and elongation comprehensively, the Al preheated to 473 K demonstrated the optimal overall mechanical properties.

**Figure 21:**
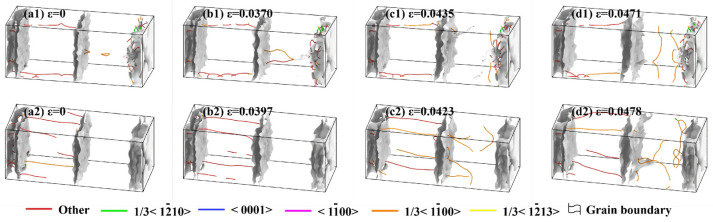
Dislocation analysis of Al/Mg bimetals at various preheating temperatures: (**a1**–**d1**) 373 K; (**a2**–**d2**) 573 K.

The fracture mechanism of the Al/Mg diffusion system with preheating and pouring temperatures of 473 and 923 K, respectively, was discussed in the previous section. This section focuses on investigating the fracture mechanism of the Al/Mg diffusion system with preheating temperatures of 373 and 473 K. The dislocation evolution characteristics of the Al/Mg bimetal preheated at 373 K and 573 K are illustrated in Figure 21. At a preheating temperature of 373 K, the fact that dislocations can cross the Al/Mg interface and subsequently multiply and glide within the Mg matrix is responsible for the observed higher fracture strain and the more gradual post−peak stress decay in the stress–strain curve. The structure at a preheating temperature of 573 K exhibits a consistent change trend with that of the curves for a preheating temperature of 473 K.

**Figure 22:**
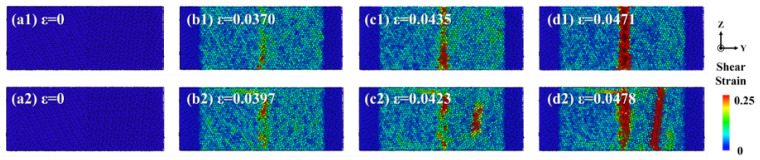
The strain distributions of Al/Mg bimetals at various preheating temperatures: (**a1**–**d1**) 373 K, (**a2**–**d2**) 573 K.

The strain distribution characteristics of the Al/Mg bimetal preheated at 573 K are presented in Figure 22. A combined analysis of the strain contour plot and DXA results revealed that, because the interfacial bonding strength is lower than the hardening limit of the matrix, this ultimately leads to interfacial fracture.

These results provide direct guidance for optimizing industrial processes by identifying that the preheating temperature exerts a more significant influence on atomic diffusion behavior than the pouring temperature. For superior performance in practical experiments, parameters should be set to a preheating temperature of 473 K and a pouring temperature of 923 K, as this combination yielded the best mechanical properties. Operators should prioritize precise control of the preheating stage to enhance atomic diffusion at the interface, subsequently utilizing the specified pouring temperature to finalize a high−quality metallurgical bond.

## 4. Conclusions

In this study, the effects of pouring and preheating temperatures on the interfacial atomic diffusion behavior and mechanical properties of the Al/Mg bimetal were investigated. The following conclusions are drawn:

(1) The influence of pouring and preheating temperature on the diffusion process exhibited consistent characteristics. Specifically, the diffusion coefficients of both Al and Mg increase with rising temperature, and the thickness of the interfacial transition layer also increases. However, the impact of preheating temperature on atomic diffusion behavior is more pronounced than the pouring temperature.
(2) The optimal mechanical properties of the Al/Mg diffusion systems were achieved at a pouring temperature of 923 K, combined with a preheating temperature of 473 K. The optimal specimen achieved a tensile strength of 1.850 GPa and an elongation of 4.51%, representing a 52.00% increase in strength and a 70.63% improvement in elongation compared to the poorest−performing sample. All the models fractured at the interface between the diffusion layer and the Mg matrix.
(3) The yielding of the structure at a pouring temperature of 1023 K is initially induced by the formation of twins, the evolution of which is subsequently accompanied by formations of numerous dislocations. In contrast, brittle fracture in the other models results directly from dislocation multiplication and slip.

The authors believe that the value of this study lies in its ability to reveal fundamental trends and underlying atomic−scale mechanisms. These insights provide direct, atomic-scale guidance for selecting the process parameters in Al/Mg bimetallic components. Additionally, they establish a foundational framework to guide the subsequent investigation into other influential factors, such as interlayers and alloying elements.

## 5. Future Work

The atomistic mechanisms identified in this study set a basis for optimizing the processing of Al/Mg bimetallic structures. The next step is experimental validation under actual fabrication conditions, utilizing the predicted optimal temperature windows (923 K for melt and 473 K for substrate preheating) to achieve strong interfacial bonding and minimize defects.

## Figures and Tables

**Table 1:** Chemical compositions of pure Al and pure Mg.

Materials	Mass Fraction(%)											
	Si	Fe	Cu	Mn	Ni	Sn	Pb	Mg	Zn	Ti	V	Al
Al	0.05	0.06	0.005	0.005	-	-	-	0.005	0.01	0.001	0.01	Bal.
Mg	0.01	0.005	0.002	0.015	0.001	0.005	0.005	Bal.	0.005	-	-	0.015

**Table 2:** The diffusion coefficients of Al atoms and of Mg atoms in the solid–solid and solid–liquid systems.

T (K)	Al				Mg			
	D (×10^−12^ m^2^/s)	D_x_ (×10^−12^ m^2^/s)	D_y_ (×10^−12^ m^2^/s)	D_z_ (×10^−12^ m^2^/s)	D (×10^−12^ m^2^/s)	D_x_ (×10^−12^ m^2^/s)	D_y_ (×10^−12^ m^2^/s)	D_z_ (×10^−12^ m^2^/s)
773 K	3.42	1.39	0.373	1.66	4.63	2.00	0.625	2.00
973 K	7.12	3.07	0.388	3.66	12.15	5.27	1.00	5.88

**Table 3:** The diffusion coefficients of Al/Mg bimetallic systems at various pouring temperatures.

T (K)	Al				Mg			
	D (×10^−12^ m^2^/s)	D_x_ (×10^−12^ m^2^/s)	D_y_ (×10^−12^ m^2^/s)	D_z_ (×10^−12^ m^2^/s)	D (×10^−12^ m^2^/s)	D_x_ (×10^−12^ m^2^/s)	D_y_ (×10^−12^ m^2^/s)	D_z_ (×10^−12^ m^2^/s)
923 K	3.89	1.72	0.248	1.92	4.65	2.25	0.41	1.99
973 K	7.12	3.07	0.388	3.66	12.15	5.27	1.00	5.88
1023 K	15.62	6.97	0.688	7.96	24.28	11.80	1.78	10.70

**Table 4:** The diffusion coefficients of Al/Mg systems at various preheating temperatures.

T(K)	Al				Mg			
	D (×10^−12^ m^2^/s)	D_x_ (×10^−12^ m^2^/s)	D_y_ (×10^−12^ m^2^/s)	D_z_ (×10^−12^ m^2^/s)	D (×10^−12^ m^2^/s)	D_x_ (×10^−12^ m^2^/s)	D_y_ (×10^−12^ m^2^/s)	D_z_ (×10^−12^ m^2^/s)
373 K	0.75	0.27	0.24	0.24	1.08	0.40	0.26	0.42
473 K	7.12	3.07	0.39	3.66	12.15	5.27	1.00	5.88
573 K	12.99	6.05	0.70	6.24	19.21	8.73	1.82	8.66

## Data Availability

The original contributions presented in this study are included in the article. Further inquiries can be directed to the corresponding authors.

## Abbreviations

The following abbreviations are used in this manuscript:

MD	Molecular Dynamics
MSD	Mean Square Displacement
RDF	Radial Distribution Function
